# The Japan hospice and palliative evaluation study 4: a cross-sectional questionnaire survey

**DOI:** 10.1186/s12904-018-0319-z

**Published:** 2018-04-20

**Authors:** Kento Masukawa, Maho Aoyama, Tatsuya Morita, Yoshiyuki Kizawa, Satoru Tsuneto, Yasuo Shima, Mitsunori Miyashita

**Affiliations:** 1 Department of Palliative Nursing, Health Sciences, Tohoku University Graduate School of Medicine, 2-1 Seiryo-machi, Aoba-ku, Sendai, Miyagi 980-8575 Japan; 2 Department of Palliative and Supportive Care, Palliative Care Team, Seirei Mikatahara General Hospital, 3453 Mikatahara-cho, Kita-ku, Hamamtsu, Shizuoka 433-8558 Japan; 3 Department of Palliative Medicine, Kobe University Graduate School of Medicine, 7-5-1 Kusunoki-cho, Chuoku, Kobe, Hyogo 650-0017 Japan; 4 Department of Human Health Sciences, Kyoto University Graduate School of Medicine, 54 Kawaharacho, Shogoin, Saikyo-ku, Kyoto, 606-8507 Japan; 5 Tsukuba Medical Center Foundation, Home Care Service, Tsukuba Medical Center Hospital, Department of Palliative Medicine, 1-3-1, Amakubo, Tsukuba, Ibaraki 305-8558 Japan

**Keywords:** Palliative care, Bereavement, Evaluation, J-HOPE study, Japan

## Abstract

**Background:**

Constant evaluation is important for maintaining and improving the quality of end-of-life care. We therefore conduct the fourth Japan Hospice and Palliative Evaluation Study (J-HOPE4) as a continuous evaluation study. In this present paper, we describe the design of J-HOPE4. The main purposes of J-HOPE4 are as follows:1) to evaluate the processes, structures, and outcomes of palliative care acute hospitals, palliative care units, and home hospice services; 2) to examine bereaved family members’ self-reported psychosocial conditions, such as grief and depression as bereavement outcomes;3) to provide data to ensure and improve the quality of care provided by participating institutions via feedback based on the results from each institution; and 4) provide clinical and academic information concerning the implications of various issues in palliative care by conducting additional studies.

**Methods:**

We will conduct a cross-sectional, anonymous, self-reported questionnaire survey. In total, 190 institutions will participate in this study, meaning that 12,000 bereaved family members will be sent a questionnaire.

**Discussion:**

This is one of the largest cross-sectional surveys involving hospice and palliative care, both in Japan and worldwide. Because this study will have a large sample size, the findings are expected to be generalizable to other settings.

## Background

End-of-life care is an important component of cancer care. Therefore, it is important to continuously measure the quality of end-of-life care to maintain and improve quality of care received by patients and their family members [1–3].

Most studies evaluating end-of-life care have been conducted with bereaved family members [4–10]. In examining end-of-life care, it can be difficult to recruit patients to studies because terminal cancer patients are often too ill to participate. Some researchers have developed valid measurements to evaluate the quality of end-of-life care from the perspective of bereaved family members such as the Quality of Death and Dying questionnaire for end-of-life [11], and the modified Quality of Death and Dying questionnaire for intensive care units [9]. Therefore, bereaved families’ survey is a useful method to evaluate end-of-life care.

The Donabedian model, “structure, process, and outcome,” is used as a framework for evaluating quality of care [12]. In Japan, Morita et al. developed the Care Evaluation Scale (CES) to evaluate the process and structure of end-of-life care [13], and Miyashita et al. developed the Good Death Inventory (GDI) to evaluate the outcome of end-of-life care [14]. These scales have been used as quality indicators of end-of-life care from the perspectives of bereaved family members in Japan.

In Japan, the initial national survey for inpatient palliative care units (PCUs) was conducted in 1997. This study developed a Satisfaction Scale for Family Members Receiving Inpatient Palliative Care, and identified factors contributing to satisfaction with perceived care [15]^.^ We have also conducted surveys on bereaved family members to evaluate the quality of end-of-life care from the perspective of family members [16, 17]. As a result, we have reported on the situation of palliative care, and obtained new knowledge about end-of-life care. The Japan HOspice and Palliative Evaluation (J-HOPE) Studies were conducted to evaluate hospice and palliative care in terms of processes, structures, and outcomes using measures such as the CES and GDI. The first J-HOPE study was conducted between 2007 and 2008, the second (J-HOPE2) 2010 and 2012, and the third (J-HOPE3) between 2013 and 2014. Table 1 shows an overview of the previous J-HOPE studies. The first J-HOPE study investigated 15 specific items including desirable methods of providing information, an evaluation of care needs, or death rattle. J-HOPE2 investigated 11 items, including sedation, social work, and desirable nursing care, and J-HOPE3 investigated 26 items including place of death, end-of-life discussions, and deathbed visions. The J-HOPE2016 was conducted as an additional survey of J-HOPE3. These four studies reported trends and important issues in hospice and palliative care in Japan. These previous J-HOPE studies contributed to the improvement of the quality of care because they revealed the necessity points for improvement [16, 17]. Additionally, feedback was provided to each participating institution so that they could compare the overall data with their own. This helped the participating institutions to review the strengths and weaknesses of their daily clinical services.

**Table 1:** Overview of J-HOPE study

	J-HOPE1	J-HOPE2	J-HOPE3
Date	May–August 2007	October 2010–April 2011	May–July 2014
Participating institution	56 designated cancer centers, 100 PCUs, 14 home hospices	20 acute hospitals, 103 PCUs, 15 home hospices	20 acute hospitals, 133 PCUs, 22 home hospices
Participants	8398 completed questionnaires for analysis: 2794 responses for designated cancer centers 5312 for PCUs 292 for home hospices	7797 completed questionnaires for analysis: 1279 responses for acute hospitals 5820 for PCUs 698 for home hospices	9126 completed questionnaires for analysis: 814 responses for acute hospitals 7294 for PCUs 1018 for home hospices
Design	Cross-sectional, anonymous, self-report questionnaire survey		
Main outcome measurements	Care Evaluation Scale-Short Version Good Death Inventory-Short Version Overall Care Satisfaction Caregiving Consequence Inventory	Care Evaluation Scale-Short Version Good Death Inventory Overall Care Satisfaction	Care Evaluation Scale-Short Version Good Death Inventory-Short Version Overall Care Satisfaction Patient Health Questionnaire 9 Brief Grief Questionnaire

We will conduct the fourth J-HOPE study (J-HOPE4) to evaluate end-of-life care in Japan. In this paper, we describe the design of J-HOPE4. The main purposes of the study are as follows: 1) to evaluate the processes, structures, and outcomes of palliative care in hospitals, PCUs, and home hospice services; 2) to examine bereaved family members’ self-reported psychosocial conditions, such as grief and depression as bereavement outcomes; 3) to provide data to ensure and improve the quality of care provided by participating institutions via feedback based on the results from each institution; and 4) to provide clinical and academic information concerning the implications of various issues in palliative care by conducting additional studies.

**Fig. 1:**
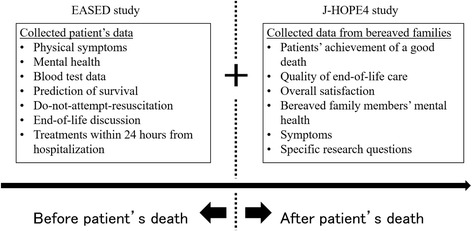
We will connect the data obtained in the EASED study and the data that will be obtained in the J-HOPE4 study. The data obtained in EASED study is on patient’s information before death. Therefore, we can discuss issues from a longitudinal perspective

This study has a number of strengths. First, we estimate that the number of participating institutions will be larger than those in the previous J-HOPE studies. Second, the number of specific researches will be larger than those in the previous J-HOPE studies. In addition, we will combine part of the data obtained in this study with that obtained in the East-Asian collaborative Study to Elucidate the Dying process (EASED) in Japan (UMIN000025457) (Fig. 1). The EASED study elucidated the dying process and end-of-life care in terminally-ill cancer patients admitted to PCUs before death. In the previous three J-HOPE studies, it was difficult to explore the causal relationship between the medical care provided before death and the quality of palliative care because they were cross-sectional surveys. Therefore, as part of this study, we can discuss these issues from a longitudinal perspective. For that reason, we expect to obtain new and valuable knowledge.

## Methods

We will conduct an anonymous, cross-sectional, self-reported questionnaire survey between May and June 2018. To identify potential subjects, we will ask each institution to identify and list up to 80 bereaved family members of patients who had died prior to January 1.

The questionnaire will be sent to the bereaved family members identified by each participating institution. A document explaining the J-HOPE4 study’s aims and procedures will be included along with the questionnaire, and the return of a completed questionnaire will be considered as consent to participate in the study. A ballpoint pen will be included in the envelope as an incentive to participate. Participants will be asked to return the completed questionnaire to the secretariat office (Tohoku University) within 2 weeks. We will send a reminder to nonresponders 1 month after sending the questionnaire. If they do not wish to participate in the study, they will be asked to check a “no participation” box and return the incomplete questionnaire. Ethical approval for the study will be granted by the institutional review boards of Tohoku University Hospital and all participating institutions.

### Participating institutions

We sent letters to 463 institutions which were approved by Hospice Palliative Care Japan (HPCJ) and included 70 acute hospitals, 337 inpatient PCUs, and 56 home hospices, and prior to July 1, 2017. Among these, 233 institutions, including 17 acute hospitals, 179 PCUs that had not participated in the EASED study (we define these institutions as ‘PCU-non EASED’), 21 PCUs that had participated in the EASED study (we define these institutions as ‘PCU-EASED’), and 16 home hospices, are going to participate in the study.

We will ask participating institutions to describe the treatment available, the bereavement care offered for family members, and the structure of the patient care provided. The structure of care in each institution includes items such as the details of religious affiliations and the numbers of medical staff members, beds, rooms, and patients. Considering the different care settings (PCUs, general hospitals, and home hospice services), we included different items to describe the institutional structure. Items concerning available treatments, such as surgery under general anesthesia, intravenous or oral chemotherapy, intravenous hydration, intravenous hyperalimentation, pleuro- and abdominocentesis, nerve block, physiotherapy/rehabilitation, and other complementary and alternative medicines, were included for PCUs and home hospices. Items concerning molecular targeted therapy, hormone therapy, radiation therapy, red-blood cell transfusion, platelet transfusion, and complementary and alternative medicines such as Maruyama and peptide vaccine hypodermic injections, thermotherapy, aromatherapy, reflexology, music therapy, lymphedema therapy by certificated specialists, and referral to available specialists, were included for PCUs. We also reviewed the institutional information available in the HPCJ database.

### Participants

The inclusion criteria are as follows: 1) the patient died of cancer, 2) the patient was aged 20 years (the age at which one is considered an adult in Japan) or older, and 3) the bereaved family member is aged 20 years or older. The exclusion criteria are as follows: 1) the patient received palliative care for less than 3 days; 2) the bereaved family member is unavailable or cannot be identified; 3) death was associated with treatment or occurred in an intensive care unit; 4) the potential participant having suffered serious psychological distress, as determined by the primary physician and a nurse; and 5) the potential participant is incapable of completing the self-reported questionnaire because of health issues such as cognitive impairment or visual disability.

### Questionnaires

Two types of questionnaires will be used in this study; main outcome (common) and specific research questionnaires. Common questionnaires will be sent to all participants, while specific research questionnaires will be randomly inserted into the documents sent to the participants. Table 2 shows the structure of the questionnaires that will be sent to the participants. Common questionnaires will include the following items and scales. The titles of the specific research are described in Table 3.

**Table 2:** Structure of questionnaire will be sent to subjects

	Common questionnaire	Specific research questionnaire	Total
Number of pages	8	4	12
Included question items or scales	• Care Evaluation Scale-Short Version • Good Death Inventory-Short Version • Overall Care Satisfaction • Patient Health Questionnaire 9 • Brief Grief Questionnaire • Symptoms patients perceived 1 week before death • Participant Characteristics	Question items from two or three specific researches selected at random

**Table 3:** List of specific studies

Title	
1. The impact of socioeconomic status on the outcomes of end-of-life care and bereavement 2. The experiences of patients and their families who had cancer of an unknown primary source 3. Care burden, turnover, and death incidences in family caregivers of cancer patients 4. Association between previous experience of end-of-life-care among family caregivers and preference of place of care and death 5. Factors that contribute to good death; perspectives form bereaved families of cancer patients 6. Relatives’ perceptions about the timing of referral and administration to hospice and palliative care units 7. Differences in perception of symptoms and the association between medical staff and caregivers and recommendations about the attitudes of medical staff 8. Non-pharmacological care preferred in the management of dyspnea for advanced cancer patients 9. Content and timing of communication between patients and their families that form the basis for end-of-life discussion; the influence on well-understood feelings and implementation of end-of-life discussion 10. Personalized symptom goal; perspectives from the bereaved family members 11. With or without chemical coping based on the judgment of the family: experiences, knowledge, and needs of the family on chemical coping 12. Bereaved family members’ preferences for timing of consultation/referral to palliative care 13. Effects of advanced care planning on its relationship and view of life and death 14. Desirable communication between cancer patients who have difficulty communicating and their families 15. Physician’s explanations about the discontinuation of aggressive anticancer therapy from the viewpoint of behavioral economics 16. Important outcomes of pharmacotherapy for dyspnea in terminal cancer patients from the perspective of the bereaved family 17. Positive effects of bereavement on the bereaved family 18. Psychological effects among families of deceased using electrocardiograph monitor within 24 h before death 19. Diagnosis of complicated grief and medical economic assessment 20. Desirable bereavement care in hospice and palliative care unit from perspective of bereaved families 21. Family experiences with terminal cancer patients with cognitive impairment 22. Effects of rehabilitation on terminal cancer patients on quality of life, and its desirable implementation 23. Families’ perceptions and needs of intravenous nutrition and hydration among advanced cancer patients 24. Coping behavior after bereavement and use of bereavement care services, and its association with grief/depression 25. Effect of family function after bereavement on depression and grief among the bereaved family 26. Bereaved family’s perceptions of immunotherapy 27. Validity of VOICES-SF Japanese version 28. Family conflicts of patients using specialized palliative care 29. Social dysfunction and labor gains/losses caused by grief after bereavement 30. Utilization of family-care leave and barriers that impede its utilization 31. Association between social distress experienced by bereaved families and social capital 32. Meaning/importance of taking a bath among Japanese terminal cancer patients 33. Spiritual pain among family caregivers of terminal cancer patients 34. Survey on cases where the place of death was not home despite transfer of place of care to the home 35. Needs in visiting nursing care and role strain in home hospice/palliative care settings 36. Chemical coping led by the family; experiences, knowledge, and needs of the family regarding to chemical coping 37. Coping behavior after the death of a loved one and use of bereaved family care services, and its association with grief/depression 38. Accuracy of recall on the patient’s symptoms, and medical practice and explanations among bereaved family 39. Does continuous deep sedation lessen communication between patients and their families? 40. Psychological outcomes of bereaved families who experience unexpected sudden death in the palliative care units 41. Bereaved families’ experiences regarding to death rattle and aspiration 42. Association between evaluation of bereaved families regarding the treatment and care for end-of-life delirium and their depression/grief/pain 43. Validity of the Good Death Scale Japanese version 44. What are the desirable death pronouncements in palliative care units? 45. Bereaved families’ opinions about pneumonia treatment 46. What symptoms and medical practices can easily cause family conflicts? 47. Influence of discussion about do-not-attempt-resuscitation with terminal cancer patients on the psychological burdens and thoughts of bereaved families 48. End of life experiences such as ‘Deathbed vision’ in palliative care units 49. Evaluation of sedation based on protocol by bereaved families 50. Quality of life of patients with malignant gastrointestinal obstruction at the end-of-life and influences on their families 51. Resilience of relatives of terminal cancer patients and its influence on their mental health 52. Association between events and care in the dying process of cancer patients and the mental health of bereaved families 53. Association between the use of ‘complementary and alternative medicines’ and depression or grief of bereaved families	

#### Overall care satisfaction

We will ask participants about their overall satisfaction with the care the patient had received at the place of death. The question asked is, “Overall, were you satisfied with the medical care the patient received?” Participants will be asked to respond using a six-point Likert scale (1: absolutely dissatisfied, 2: dissatisfied, 3: somewhat dissatisfied, 4: somewhat satisfied, 5: satisfied, and 6: absolutely satisfied).

#### Care evaluation scale-short version

The CES was developed to measure end-of-life care from the perspective of bereaved family members, with a focus on the structure and process of care. The original version of the CES includes 10 domains and 28 attributes. The questionnaire was designed to ensure that respondents evaluated the structure and process of end-of-life care by rating the need for improvement for each item on a six-point Likert scale (1: improvement is highly necessary; 2: improvement is quite necessary; 3: improvement is necessary; 4: improvement is somewhat necessary; 5: improvement is slightly necessary; and 6: improvement is not necessary). Total scores will be transferred to a 100-point scale, with higher scores indicating better care. The short version of the CES consists of 10 representative items from each domain, and the validity and reliability of the scale have been confirmed [13]. We will use the revised short version of the CES (CES2) in the current study [18].

#### Good death inventory-short version

We will use the short version of the GDI to measure patients’ achievement of a good death from the perspective of bereaved family members. The original version of the GDI consists of 10 core and 8 optional domains and 54 attributes. The 10 core domains evaluate the attributes that Japanese people consistently rate as important, and the 8 optional domains evaluate attributes that are rated as important, albeit inconsistently, and depend upon individual values [14]. The short version of the GDI consists of 18 representative items from each domain, and the validity and reliability of the scale have been confirmed. Participants will evaluate each attribute using a seven-point Likert scale (1: absolutely disagree, 2: disagree, 3: somewhat disagree, 4: unsure, 5: somewhat agree, 6: agree, and 7: absolutely agree). The total score will be calculated by summing the scores for all attributes, with a high total score indicating the achievement of a good death.

#### Brief grief questionnaire

We will use the Brief Grief Questionnaire (BGQ) to assess complicated grief (CG). The BGQ was developed by Shear et al., and the reliability and validity of the Japanese version have been confirmed [19]. Although the BGQ was originally developed to assess CG in people who had lost a loved one to, in the September 11 attacks, Fujisawa et al. used the questionnaire with the general Japanese population including bereaved individuals who had lost a loved one to cancer [20]. A total score of 8 or higher indicates that the respondent is likely to develop CG, scores of 5–7 indicate subthreshold CG, and scores of < 5 indicate that the respondent is unlikely to develop CG.

#### Patient health questionnaire 9

The Patient Health Questionnaire 9 is a widely accepted instrument that consists of 9 items used to assess depressive symptoms. This instrument is widely used as a brief diagnostic tool and measures the severity of depression in both clinical practice and research; the reliability and validity of the scale have been confirmed [21–23]. Each of the nine items concerns the extent to which a particular depressive symptom has bothered the respondent in the preceding 2 weeks. Responses are provided on a scale ranging from 0 (not at all) to 3 (nearly every day), and total scores range from 0 to 27. Scores of 5, 10, 15, and 20 represent valid cutoff points representing minimal, mild, moderate, moderately severe, and severe depression [21].

#### Symptoms

We will ask the bereaved family members to report physical symptoms that the patients experienced 1 week before death. If an eligible patient had experienced a symptom, the bereaved family will describe the degree of symptom severity. Responses will be provided on a scale ranging from 0 (not at all) to 4 (very acute). The targeted symptoms are: 1) pain; 2) fatigue; 3) nausea; 4) constipation; 5) anorexia; 6) weight loss; 7) drowsiness; 8) insomnia; 9) dyspnea; and 10) itching.

### Data analysis

#### Expected sample size

Since this research is a questionnaire survey, the expected number of questionnaires to be sent was considered based on accuracy. We estimate that 20 acute hospitals, 150 PCUs, and 20 home hospice services will participate in the study because about 180 institutions participated in J-HOPE3 and the number of institutions approved by HPCJ has increased. If each institution identifies 80 participants, then 12,000 participants at PCUs, 1600 at acute hospitals, and 1600 at home hospices will be identified. Therefore, the total number of expected participants is about 15,000. The number of responses eligible for analysis is expected to be about 9750 because we estimate that the response rate will be about 65% in accordance with the previous J-HOPE studies.

#### Main statistical analyses

We will calculate the mean and distribution of each outcome measurement by institution. We will perform univariable and multivariable analyses using outcome measurements as objective variables. Therefore, we will reveal the factors associated with outcomes such as GDI and CES.

In terms of specific research, each principle investigator calculated sample size, and will analyze data according to each research plan.

## Discussion

This paper outlines the study protocol of J-HOPE4. The main objective is to evaluate the processes, structures, and outcomes of palliative care in acute hospitals, PCUs, and home hospice services. This is one of the largest cross-sectional surveys involving hospice and palliative care, that has been conducted both in Japan and worldwide. This study has several strengths and limitations.

### Strengths

We think our study has several strengths. First, the sample size is large. Therefore, the findings are expected to be generalizable to other settings. Second, because this study includes many specific researches, we are likely to obtain useful knowledge for clinical practice.

### Limitation

The participants are those who lost a loved one in a HOPCJ member facility in Japan. Therefore, the findings of this study may not be generalizable to other countries. In addition, there may be recall bias because of the retrospective nature of the study. However, according to some studies, considering both recall bias and grieving process, 3–12 months after death may be an appropriate time frame for participant inclusion [24–26].

## Abbreviations

BGQ: the Brief Greif Questionnaire
CES: the Care Evaluation Scale
CG: complicated grief
EASED: the East-Asian collaborative Study to Elucidate the Dying process
GDI: the Good Death Inventory
HPCJ: the Hospice Palliative Care Japan
J-HOPE: the Japan Hospice and Palliative Evaluation
PCUs: palliative care units
